# The TAB1-p38**α** complex aggravates myocardial injury and can be targeted by small molecules

**DOI:** 10.1172/jci.insight.121144

**Published:** 2018-08-23

**Authors:** Gian F. De Nicola, Rekha Bassi, Charlie Nichols, Mariana Fernandez-Caggiano, Pelin Arabacilar Golforoush, Dibesh Thapa, Rhys Anderson, Eva Denise Martin, Sharwari Verma, Jens Kleinjung, Adam Laing, Jonathan P. Hutchinson, Philip Eaton, James Clark, Michael S. Marber

**Affiliations:** 1 British Heart Foundation Centre of Excellence, The Rayne Institute, St. Thomas’ Hospital, and; 2 The Randall Division, New Hunt’s House, Guy’s Campus, King’s College London, United Kingdom.; 3 Bioinformatics Facility, The Francis Crick Institute, London, United Kingdom.; 4 Department of Immunobiology, King’s College London, United Kingdom.; 5 Platform Technologies and Science, GlaxoSmithKline, and; 6 Discovery Partnerships with Academia, GlaxoSmithKline, Medicines Research Centre, Stevenage, United Kingdom.

**Keywords:** Cardiology, Therapeutics, Pharmacology, Protein kinases, Structural biology

## Abstract

Inhibiting MAPK14 (p38α) diminishes cardiac damage in myocardial ischemia. During myocardial ischemia, p38α interacts with TAB1, a scaffold protein, which promotes p38α autoactivation; active p38α (pp38α) then transphosphorylates TAB1. Previously, we solved the X-ray structure of the p38α-TAB1 (residues 384–412) complex. Here, we further characterize the interaction by solving the structure of the pp38α-TAB1 (residues 1–438) complex in the active state. Based on this information, we created a global knock-in (KI) mouse with substitution of 4 residues on TAB1 that we show are required for docking onto p38α. Whereas ablating p38α or TAB1 resulted in early embryonal lethality, the TAB1-KI mice were viable and had no appreciable alteration in their lymphocyte repertoire or myocardial transcriptional profile; nonetheless, following in vivo regional myocardial ischemia, infarction volume was significantly reduced and the transphosphorylation of TAB1 was disabled. Unexpectedly, the activation of myocardial p38α during ischemia was only mildly attenuated in TAB1-KI hearts. We also identified a group of fragments able to disrupt the interaction between p38α and TAB1. We conclude that the interaction between the 2 proteins can be targeted with small molecules. The data reveal that it is possible to selectively inhibit signaling downstream of p38α to attenuate ischemic injury.

## Abstract 2

Disrupting TAB1-p38α interaction in vivo has a protective effect during myocardial ischemia and can be achieved in vitro with small molecule inhibitors.

## Introduction

The MAPK p38α is a serine threonine protein kinase that is activated by stress. The kinase is expressed in all mammalian cell types and is highly conserved in yeast. Ablating p38α in mice results in early embryonal lethality, and in yeast, it results in failures in adaptation to environmental stress and pheromones. Hence, p38α has an essential role in health and homeostasis (1, 2).

Nonetheless, in animal models of acute myocardial infarction and heart failure, inhibition of p38α reduces infarct size, improves left ventricular function, and limits cardiac dilatation. Conversely, cardiac-restricted forced activation of p38α damages the myocardium. In human hearts, p38α is activated by myocardial ischemia. Thus, p38α inhibition makes therapeutic sense in ischemic heart disease (3–5).

The clinical trials that have been published to date, those that are on-going, and those that are planned all use pharmacological inhibitors of p38 kinase activity that target the ATP binding site and inhibit the catalytic activity of the kinase in all settings. In all cases where information is available, the clinical trials have been suspended due to adverse side effects such as skin and liver toxicity. The adverse effects are shared between inhibitors based on different scaffolds and with diverse modes of binding to the ATP binding site of p38 (3). Based on the biological importance of this kinase, its ubiquitous expression, and the shared toxicity profile of diverse inhibitors, the assumption is that the side effects are a manifestation of on-target toxicity. Consequently, current trials are carefully designed to achieve only partial p38 inhibition at peak concentration. Despite this care in the controlled environment of a clinical trial, liver toxicity is still evident, even in dosing regimens that have been carefully titrated or compromised to cause only partial or suboptimal inhibition. Thus, p38 inhibition is attractive in heart disease; however, an alternative strategy is needed, whereby circumstance-specific inhibition of p38α is achieved (6, 7). A number of groups have shown that, during myocardial ischemia, TAB1 binds to and induces autoactivation of p38α, which in turn phosphorylates TAB1. In other words, TAB1 is both an activator and a substrate of p38α. This mechanism of activation is independent of MAP2K3 and MAP2K6, 2 archetypical upstream activators of p38α. TAB1 is a scaffold protein, the N-terminal region (residues 1–370) is a pseudo phosphatase domain with no catalytic activity, and the C-terminal region is unstructured and contains a p38α and a TAK1 activating region (8, 9). We have previously solved the X-ray structure of p38α in complex with TAB1 (residues 384–412) peptide, identified the TAB1 binding site on p38α, and showed the structural rearrangements within p38α induced by TAB1 (residues 384–412) that cause autoactivation (8, 10–12). Here, we investigated the role of p38α and TAB1 phosphorylation on the interaction by solving the X-ray structure of the complex between p38α and TAB1 (residues 1–438) in their phosphorylated forms. The structural data suggest that TAB1 does not dissociate from p38α after inducing autoactivation and becoming phosphorylated. To examine the physiological relevance of the p38α-TAB1 complex, we generated a global knock-in (KI) mouse where 4 single point mutations on the regions of TAB1 responsible for the interaction were mutated and prevented TAB1 from docking onto p38α. We used the mouse model to elucidate the role of the interaction during myocardial ischemia in vivo — a setting where p38α is known to be activated through the TAB1 interaction and to cause TAB1 phosphorylation (10). In the KI mice, the myocardial infarction volume as a percentage of the risk volume was significantly reduced, and the ability of active p38α to phosphorylate TAB1 was limited; however, we did not observe a major impairment of p38α activation. Data in vitro suggest that persistent p38α activation may be the result of the disinhibition of MAP2K3 access in the absence of TAB1 docking on p38α. Finally, through a fragment screen, we identified a group of small molecules, the adamantanes, which bind to p38α and interfere with TAB1 recognition. This demonstrated that the interaction can be targeted by small molecules. Based on the results presented here, we propose that TAB1 phosphorylation downstream of p38α is associated with cardiac damage during ischemia. Moreover, disabling this mode of activation through the germline didn’t cause any discernible phenotype, suggesting a path to avoid the toxicity observed with ATP competitive inhibitors of p38α

## Results

### The effect of phosphorylation on the p38α-TAB1 interaction

TAB1 binds to p38α and induces autophosphorylation of residues Thr180 and Tyr182; active p38α is then able to phosphorylate TAB1 on residue Serine 423 (13). We wanted to test whether phosphorylation of either protein would modulate their binding affinity and/or the surface they use to interact with each other. To this end, we produced phosphorylated p38α (pp38α) and phosphorylated TAB1 at Serine 423 (p^S423^TAB1), measured their binding affinity, solved the X-ray structure of the complexes, and compared these to our previously published data on nonphosphorylated p38α and a short TAB1 (residues 384–412) 29-mer peptide (8).

### Thermodynamic characterization of the binding between pp38α-p^S423^TAB1, p38α-TAB1, and pp38α-TAB1

**Figure 1:**
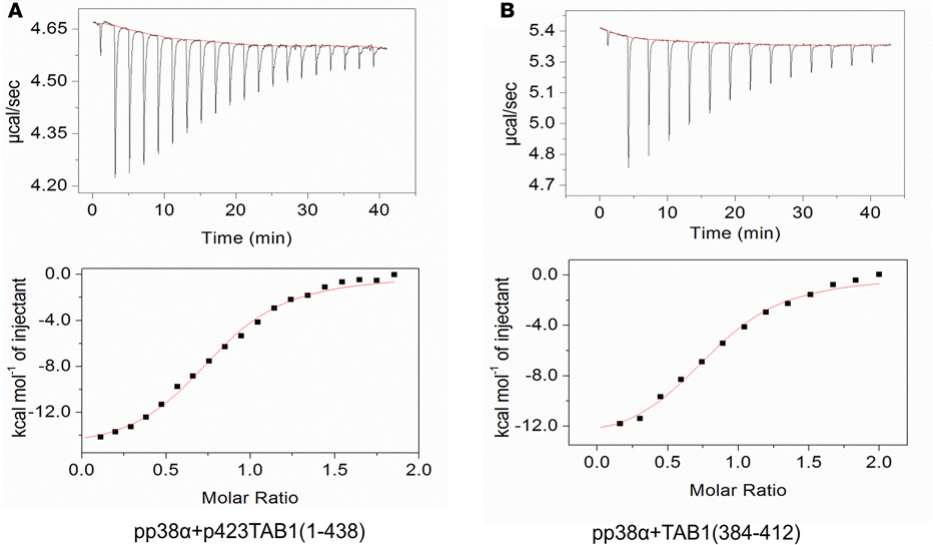
Thermodynamic characterization of the interaction between p38α and TAB1 in their phosphorylated and nonphosphorylated forms. Top panels: ITC raw data of the interaction between dual (pThr180, pTyr182) phosphorylated p38α (pp38α) and TAB1 in mono (pSer423) phosphorylated (**A**) and native forms (**B**). Bottom panels: all but the first titration point were used for the curve fitting.

The binding profiles of the interaction between p38α and TAB1, pp38α and TAB1, and pp38α and p^S423^TAB1 were very similar, irrespective of their phosphorylation state. The conclusion is that phosphorylation does not affect the binding affinity between p38α and TAB1 (Figure 1 and Table 1).

### Comparing p38α-TAB1 and pp38α-TAB1 X-ray structures

**Figure 2:**
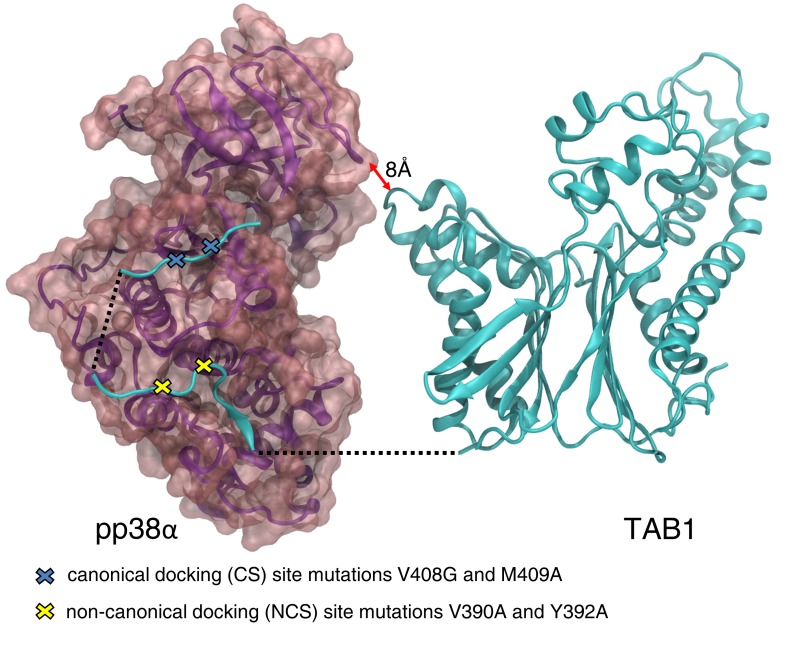
X-ray structure of the dual phosphorylated pp38α-TAB1 (residues 1–438) complex. TAB1 is shown in ribbon format (cyan) and pp38α in ribbon format (magenta) with a transparent surface overlay. The TAB1 residues belonging to the pseudo PP2 domain of TAB1 (residues 15–370) and the residues interacting with pp38α are visible. The N- (residues 1–14) and C- (residues 413–438) terminal regions, the residues (residues 371–383) between the pseudo PP2 domain and the p38α binding region and the residues (residues 396–402) between the canonical and noncanonical site are disordered and not visible in the X-ray structure. The conclusion is that no other region beyond residues 384-412 of TAB1 interacts with p38α, confirming the ITC data of thermodynamically similar binding characteristics between TAB1 (residues 1–438) and TAB1 (residues 384–412).

**Figure 3:**
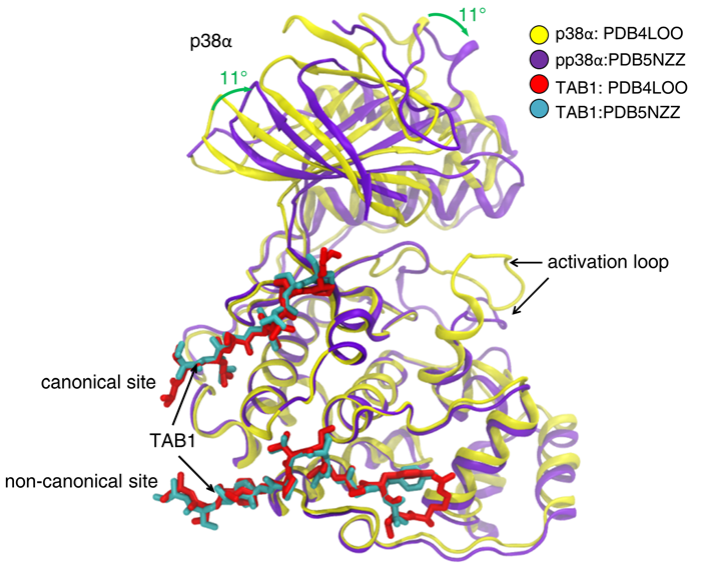
Comparison of p38α bound to TAB1 peptide (residues 384–412, PDB 4LOO) and pp38α bound to TAB1 protein (residues 1–438, PDB 5NZZ). Activation of p38α induces a major shift in the conformation of the activation loop and the N-terminal domain rotates approximately 11° toward the C-terminal domain creating a more closed active site typical of activated kinases. The rest of the C-terminal domain of p38α and the p38α interacting region of TAB1 align closely, which is consistent with the observation that the affinity of TAB1 for p38α and pp38α is virtually identical.

The X-ray structures of pp38α and nonphosphorylated p38α with TAB1 showed that the 2 complexes used the same binding interface to interact with one another. The phosphorylation/activation status of p38α had no material effect on the complex. TAB1 bound to the C-terminal lobe of p38α in a bipartite manner: residues 384–394 of TAB1 bound to a hydrophobic pocket created by α_F_ and α_H_ helices and the loop connecting helix α_2L14_ to helix α_H_ (this site is defined as the noncanonical docking site [NCS] because it is specific to TAB1), and residues 404–412 of TAB1 bound within the groove defined by α_D_ and α_E_ helices and the reverse turn between β_7_ and β_8_ (this site is defined as the canonical docking site [CS] because it is also used by other substrates and activators of p38α, such as MAP2K3 and MEF2A) (Figure 2). Phosphorylation did not affect the binding interface between p38α and TAB1; it instead induced major conformational changes of the activation loop of p38α, which contains the phosphorylation sites Thr180 and Tyr182 (Figure 3). In p38α-TAB1 complex, TAB1 binding positioned the activation loop of p38α proximal to the ATP binding site, primed for the autophosphorylation reaction. This was in keeping with the fact that the phosphorylation loop is the substrate of the intramolecular autoactivation reaction. In pp38α-TAB1 complex, upon phosphorylation, the now-activated loop of the kinase swung away from the ATP binding site, freeing access to the catalytic site for other substrates.

**Figure 4:**
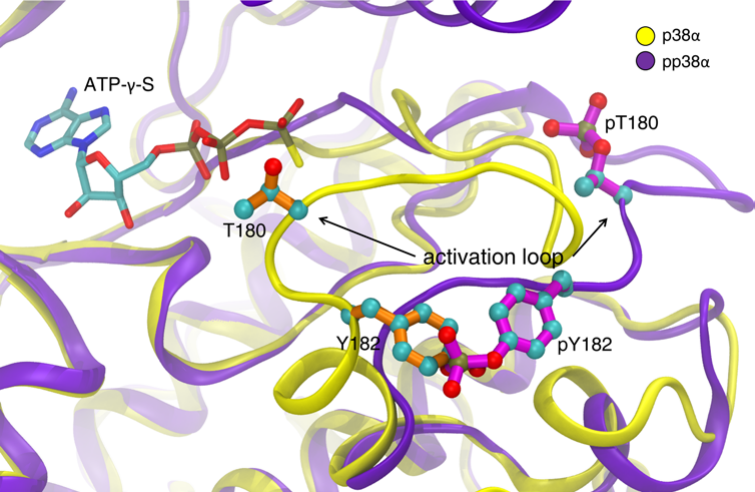
Comparison of p38α and pp38α activation loops in the complexes. This comparison illustrates the reorientation of Y182 and movement of T180 away from the ATP binding site upon phosphorylation. For clarity, only the ATP-γ-S ligand of the pp38α structure is shown.

In pp38α, residue pThr180 sat at the interface between the 2 domains of the kinase with the Thr180 phosphate group interacting with Arg67, Arg70, and Lys66 from the N-lobe and Arg149 and Arg173 from the C-lobe, whereas the phosphate from pTyr182 interacted with Arg186 and Arg189 on the C-lobe. This loop arrangement is typical of active kinases (Figure 4).

The crystallographic and biophysical data both suggest that TAB1 remains bound to p38α after having induced the kinase autoactivation and having been phosphorylated by it; they also show that no other region, beyond residues 384–412 in TAB1, was involved in the interaction with p38α.

### Assessing the effect of TAB1 docking-site mutations on its ability to induce p38α autophosphorylation and to act as a substrate of p38α in bacterial and mammalian systems

**Figure 5:**
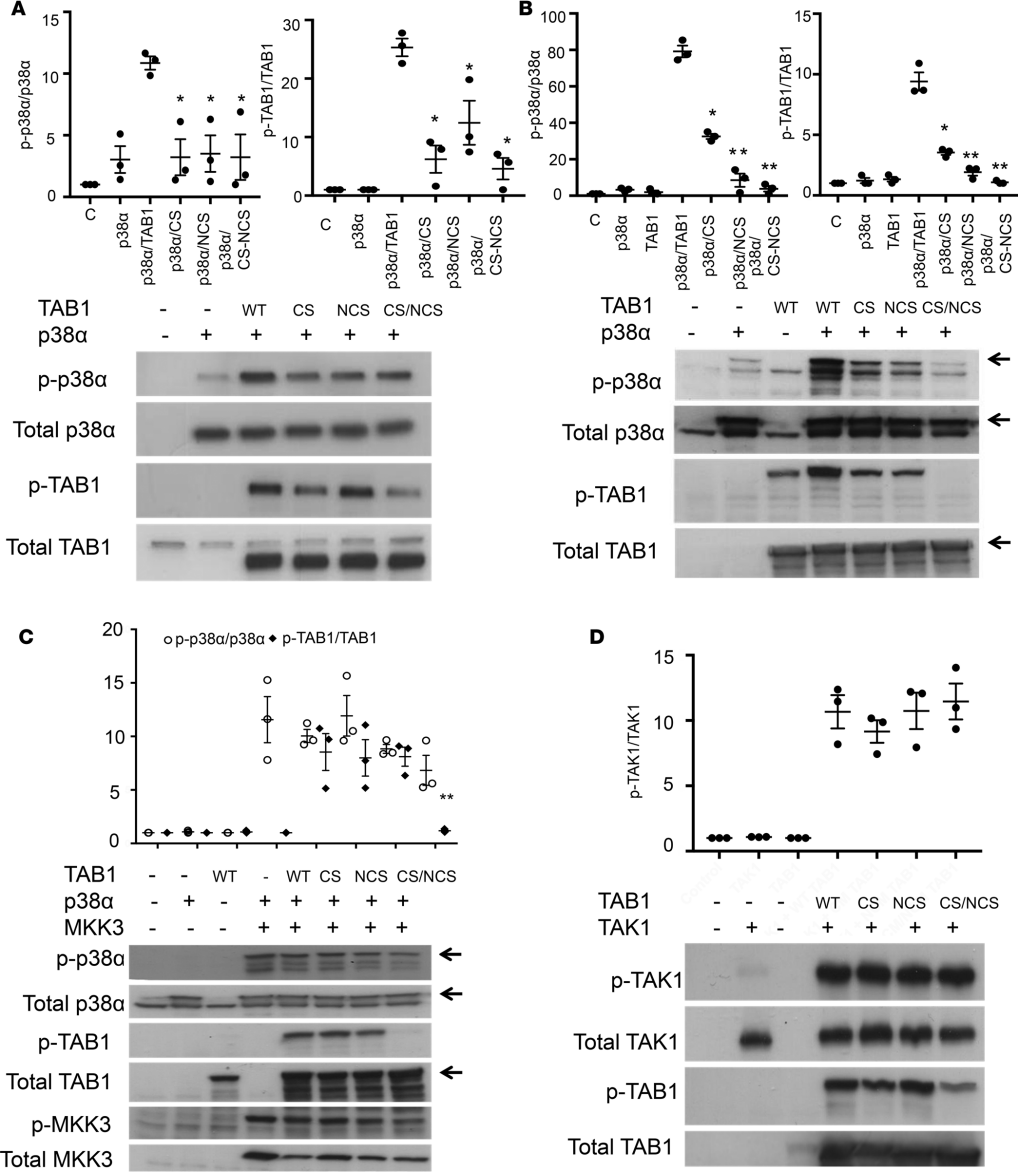
Characterizing the interaction between p38α and wild type (WT) and mutated forms of TAB1 by co-expression. (**A**) Overexpression of p38α and TAB1 in *E.Coli*. Coexpression of TAB1 and p38α increases p38α and TAB1 phosphorylation. Mutation of each of the individual recognition sites in TAB1 diminishes these phosphorylations with the strongest effect apparent when both sites are mutated. (**B**) Similar results are obtained with overexpression of p38α and TAB1 in HEK293. (**C**) Overexpression in HEK293 cells of p38α, TAB1, and MKK3b in an attempt to normalize the levels of p38α activation. The data show that the recognition regions within TAB1 that are needed to autoactivate p38α are also those used to present TAB1 as a p38α substrate. (**D**) Stopping TAB1 from docking onto p38α does not affect the ability of TAB1 to activate TAK1. One-way ANOVA was used for the statistical analysis, *P < 0.05, **P < 0.01 vs. p38α/TAB1 WT.

TAB1 is a substrate of p38α, as well as an activating binding partner (9). Mutants of TAB1 with residue substitutions in each of the 2 regions responsible for p38α binding (V390A and Y392A in the NCS and V408G and M409A in the CS) were created in the full-length TAB1 (residues 1–502) to examine the ability of these mutants to induce autophosphorylation of p38α and of pp38α to transphosphorylate the mutants in mammalian and bacterial systems. Following IPTG induction and recombinant expression of p38α and WT and mutant TAB1 proteins in transformed *E.coli*, phosphorylation of p38α and TAB1 were analyzed by immunoblotting. WT TAB1 increased the phosphorylation of p38α, and this was reduced by mutations within TAB1. This pattern was reflected in TAB1 phosphorylation, with the lowest phosphorylation evident on the double mutant (Figure 5A). To complement this, a similar investigation was performed in mammalian cells (HEK293), with similar findings (Figure 5B). The interpretation of the results is, however, complicated by the interrelation between the ability of TAB1 to activate p38α and also to become phosphorylated by p38α. In other words, is the reduced phosphorylation of the CS/NCS mutant the result of its inability to activate p38α or its inability to dock on p38α? To answer this question, we coexpressed MKK3b, the most abundant splice variant of MAP2K3, to equally activate p38α in the presence of the different TAB1 mutants in a mammalian setting. This confirmed that the recognition regions within TAB1 needed to autoactivate p38α are also utilized to recognize it as a p38α substrate (Figure 5C). The in vivo cellular and the in vitro biophysical data are, therefore, in agreement: the TAB1 V390A/Y392A/V408G/M409A substitutions disabled p38α autoactivation and the ability of p38α to transphosphorylate TAB1. The question is whether they have similar effects on other TAB1 binding partners?

### Assessing the effect of TAB1 mutation on activation of TAK1

TAB1 is also a binding partner of TAK1, an upstream kinase in the p38α activation pathway. TAB1 is able, by using 2 distinct C-terminal regions, to induce auto-activation of p38α and TAK1. Previous reports suggest that TAB1 phosphorylation acts as a negative feedback by inhibiting TAB1-induced TAK1 autophosphorylation and subsequent p38α phosphorylation (13). Firstly, contrary to previous reports, in HEK293 cells, we could not clearly show that TAB1 phosphorylation alters TAK1 autophosphorylation. Then we examined if the mutations within TAB1 similarly affect its ability to autophosphorylate TAK1. The mutant forms of TAB1, with diminished ability to induce autophosphorylation of p38α, were still able to efficiently activate TAK1. We concluded that the genetic inhibition of the p38α-TAB1 interaction does not perturb TAK1 signaling (Figure 5D).

### The generation of the TAB1 (V390A, Y392A, V408G, and M409A) KI mouse

**Figure 6:**
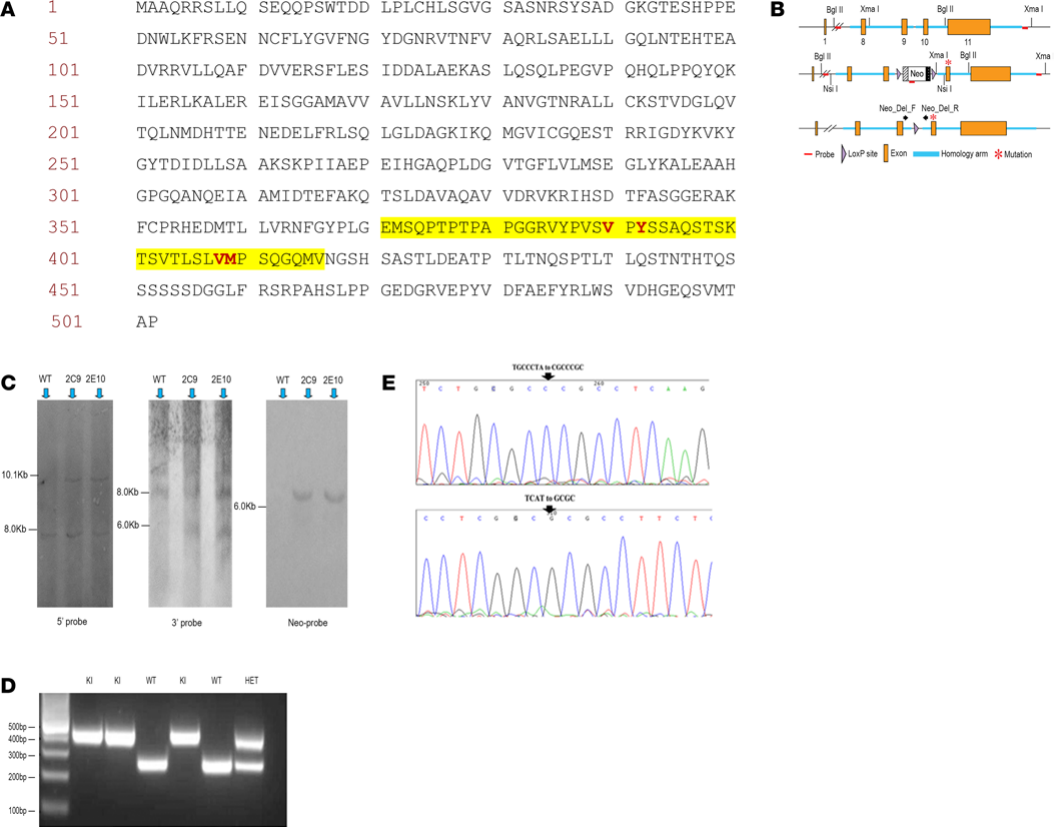
Creation of the TAB1 (V390A,Y392A,V408G and M409G) KI mouse. (**A**) TAB1 protein sequence, the targeted residues are shown in red. (**B**) Targeting strategy to produce the TAB1 (V390A, Y392A, V408G, and M409A) KI mouse. Schematic representation of the WT allele, targeted allele, and the constitutive KI allele after Cre recombination. (**C**) Southern blot of WT and 2 targeted ES cell clones (denoted as 2C9 and 2E10) with 3 probes. The expected molecular weight band for WT and the targeted allele are shown. (**D**) PCR of the targeted allele using genomic DNA derived from tail snips. (**E**) Sequencing of the genomic DNA of the targeted allele confirming the 4 single point mutations.

p38α or TAB1 global KOs are embryonal lethal, and based on the X-ray structures of the protein complexes in their active and nonactive form, we decided to create a global KI mouse with residue substitutions in each of the 2 TAB1 regions responsible for p38α recognition (V390A and Y392A in the NCS and V408G and M409A in the CS) (1, 14). The 4 point mutations introduced in the TAB1 gene prevented TAB1 from docking onto p38α (Figure 6A and Figure 2 show the position of the mutations on the TAB1 primary sequence and on the quaternary structure of the complex, respectively). The targeting construct was made using a bacterial artificial chromosome (BAC) containing the mTab1 gene (GenBank accession number: NM_025609.2, Ensembl: ENSMUSG00000022414, https://www.ensembl.org/Mus_musculus/Gene/Summary?db=core;g=ENSMUSG00000022414;r=15:80133127-80161707). The gene is located on mouse chromosome 15; it contains 11 exons with the ATG start codon in exon 1 and TAG stop codon in exon 11. The amino acid residues V390, Y392, V408, and M409 are located in exon 10. The targeting vector 5′ homology arm and 3′ homology arm was amplified from BAC DNA and confirmed by end sequencing; the mutations V390A, Y392A, V408G, and M409A were introduced into exon 10 by site-directed mutagenesis. The constitutive KI allele was obtained after Cre-mediated recombination, and C57BL/6 ES cells were used for gene targeting (Figure 6B). The ES cells bearing the integrated construct as determined by Southern blot (Figure 6C) were introduced into C57BL/6J mouse blastocysts and then transferred to a pseudopregnant female. The genomic DNA extracted from the tail clips of PCR (Figure 6D) positive offspring was sequenced to confirm the presence of the V390A, Y392A, V408G, and M409A mutations (Figure 6E).

### Baseline characterization of the TAB1 (V390A, Y392A, V408G, and M409A) KI mouse

**Figure 7:**
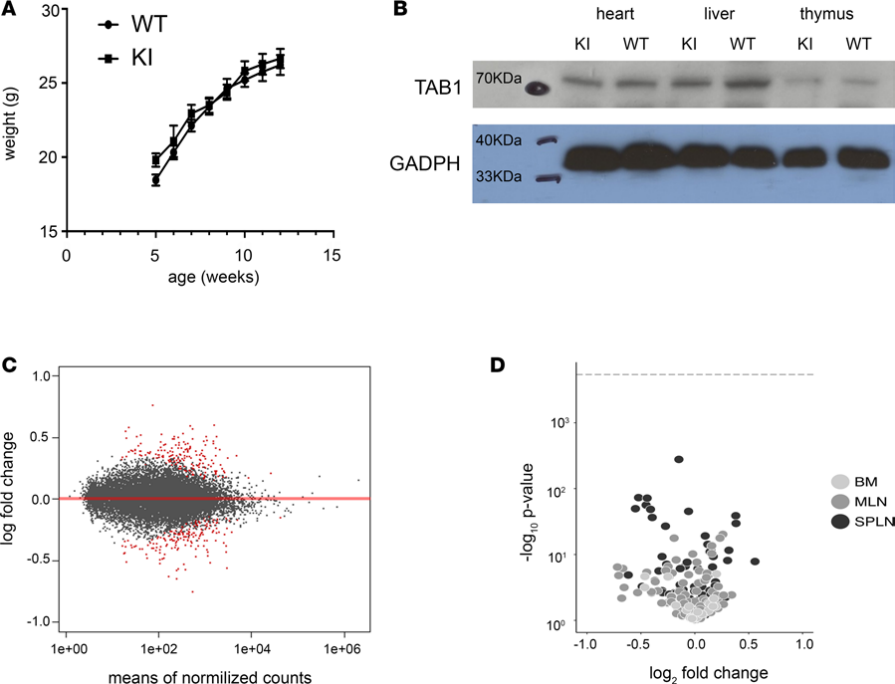
(A) Growth curves comparing within colony WT and KI mice. Mouse weights taken between 5 and 12 weeks of age from each genotype were analyzed using 2-way repeated measures ANOVA. Weights are not significantly different. (**B**) Immunoblot analysis of total TAB1 expressed in organs of WT vs. KI mice. (**C**) Volcano plot showing minimal changes of the transcriptome profile between WT and KI hearts. (**D**) Immune phenotype characterization. Each data point represents a cell subset. MLN, mesenteric lymph nodes; SPLN,spleen. The position of the data point on the *x* axis represents how the mean size of that subset changes in the KI relative to the WT. The position of each data point on the *y* axis represents the 1/*P* value. The dotted horizontal line represents the Bonferroni adjusted threshold for statistical significance. None of the populations are statistically different between KI and WT mice.

The homozygous KI mice were viable, expressed similar amounts of the mutated form of TAB1 as in-colony WT mice, and developed normally (Figure 7, A and B). The baseline characterization shows that the global inhibition of p38α-TAB1 complex formation, in all tissues and through all stages of development, had no obvious consequence. The question is whether more subtle effects exist.

### Targeting TAB1 (V390A, Y392A, V408G, and M409A) does not affect the cardiac transcriptome or the immune cell repertoire

#### Transcriptomic profile of the left ventricle.

We tested whether there were significant transcriptional consequences to expressing TAB1 (V390A, Y392A, V408G, and M409A) in mouse left ventricular myocardium. We performed RNA-sequence (RNA-Seq) analysis of KI versus WT heart and observed no major transcriptional differences. The highest log_2_-fold change detected was 0.75; in fact, only 27 genes out of the approximately 14,000 detected showed a log_2_-fold difference in either direction of greater than 0.5. All other genes showed fold differences below 0.5. When the list of differentially expressed genes with a *P* adjusted value below 0.05 is analyzed through the Reactome database pathway (https://reactome.org/); the only biological pathway that shows a significant overrepresentation in the list of genes is the circadian clock system. Finally, none of the known genes involved in p38α signaling were differentially expressed (15).

Our interpretation is that, at baseline, beyond the circadian clock pathway, there is no significant biological difference in the transcriptome profile of the WT vs. KI hearts (Figure 7C and Table 2).

### Immunophenotype

p38α is expressed in, and regulates the function of, several classes of immune cells. Of most relevance, p38α autophosphorylation plays a role in T lymphocyte maturation, including through TAB1 (16, 17). We therefore examined and compared the repertoire of immune cell populations in the spleen, BM, and mesenteric lymph nodes in WT vs. KI mice (Figure 7D). These analyses did not reveal any statistically significant differences in macrophage or lymphocyte abundance or maturation.

### Myocardial ischemia and infarction in TAB1 (V390A, Y392A, V408G, and M409A) KI mice

Ischemia activates p38α, and pharmacological inhibition of p38α during ischemia reduces heart damage. Our group has previously shown that, during ischemia, p38α is activated through autophosphorylation induced by the interaction with the scaffold protein TAB1 (8, 11, 12). Here, we subjected WT and KI mice to in vivo regional ischemia to measure the activation of p38α, TAB1 phosphorylation, and subsequent infarct size.

**Figure 8:**
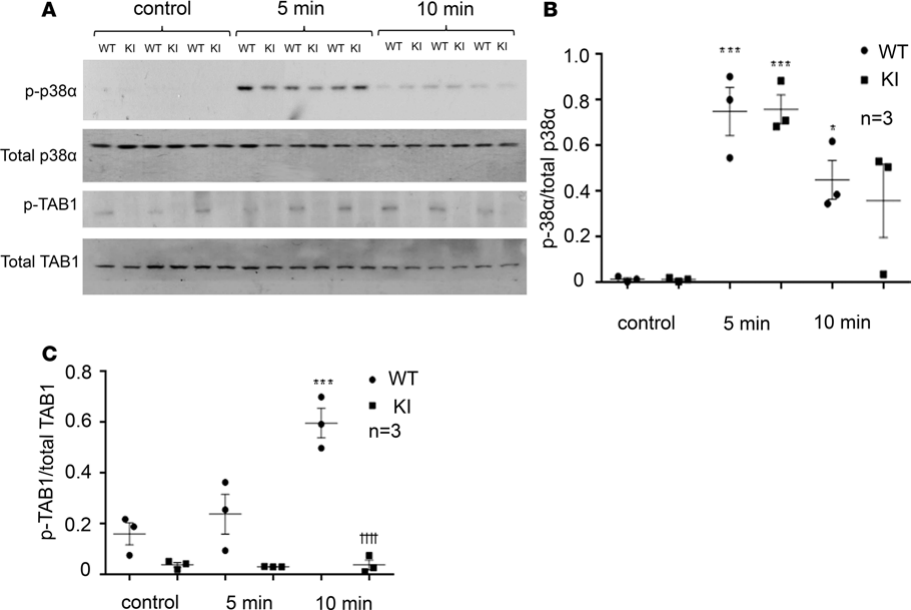
(A) Immunoblot analysis of heart homogenates taken from hearts subjected to regional myocardial ischemia at 5 and 10 minutes. (**B**) Quantification of phosphorylated p38α normalized against total p38α. WT control vs. WT and control vs. KI at 5 minutes, ***P < 0.001; WT control vs. WT at 10 minutes, *P < 0.05. (**C**) Phosphorylated TAB1 normalized against total TAB1. WT control vs. WT 10 minutes, ***P < 0.001; WT vs. KI at 10 minutes, ††††*P* < 0.0001. As previously reported in the literature, ischemia causes p38α activation and subsequent TAB1 phosphorylation in the WT hearts. In the KI heart, TAB1 phosphorylation is almost completely abolished during ischemia, despite the nearly equivalent activation of p38α.

As previously reported in the literature, ischemia causes p38α activation and TAB1 phosphorylation (Figure 8) (18, 19). We did not observe significant differences in the levels of p38α activation between WT and KI hearts at 5 minutes. At 10 minutes, there appeared to be slightly less p38α activation in KI hearts, but the magnitude of this difference was small (Figure 8B). In contrast, the amount of TAB1 phosphorylation was strikingly and significantly lower in the KI hearts. Thus, while p38α was activated in the KI hearts, it was unable to transphosphorylate TAB1 (Figure 8C). We concluded that, in the KI hearts, a compensatory mechanism is in place, which allows p38α activation despite inhibition of p38α-TAB1 complex formation.

**Figure 9:**
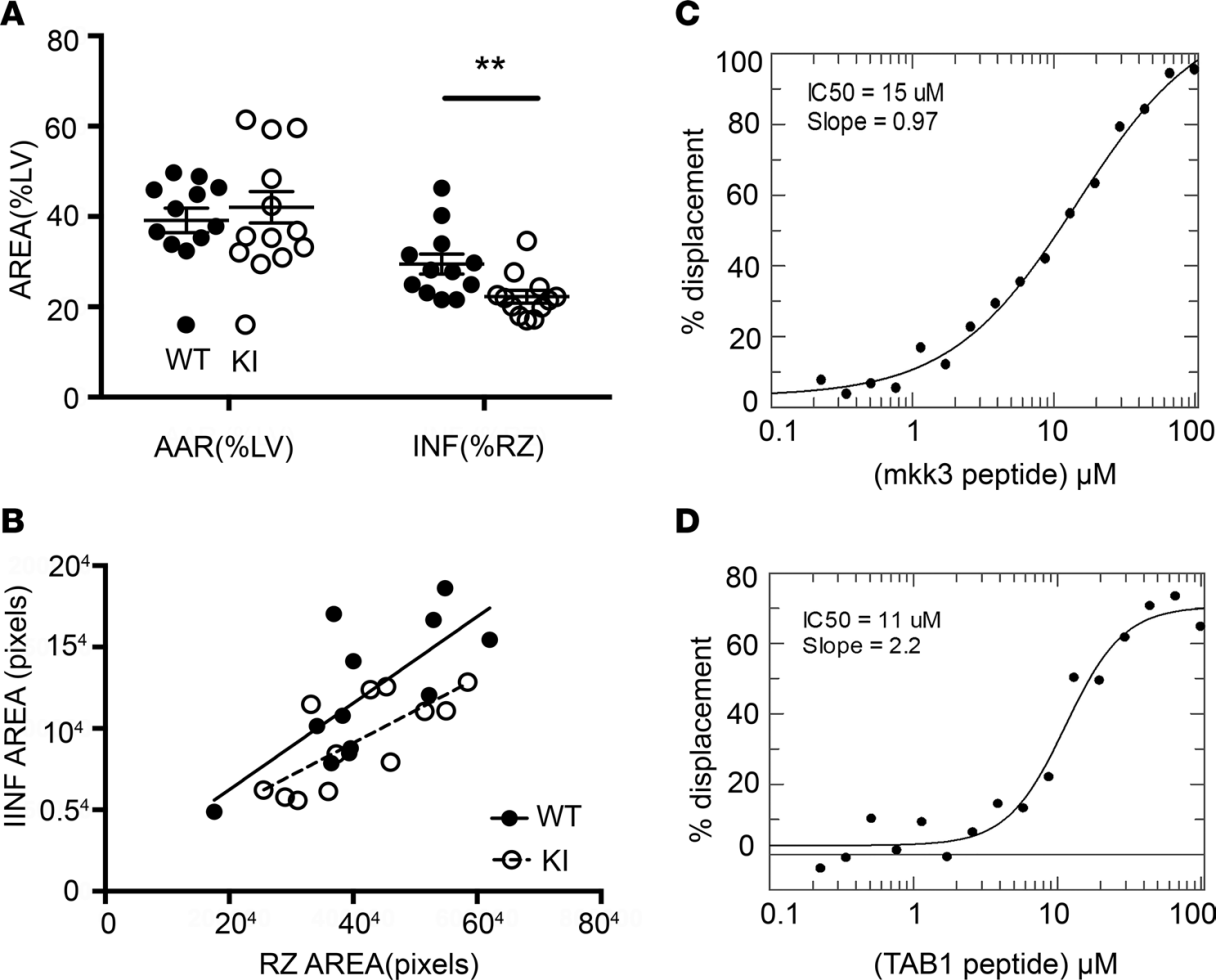
Myocardial infarction in TAB1 WT and KI mice, and competition between TAB1 and MKK3b on p38α. (**A**) Myocardial infarction volume (INF) as a percentage of the risk zone (RZ) in the area at risk (AAR) of the left ventricle (LV) is significantly reduced in KI mice after 30 minutes of occlusion of the left anterior descending coronary artery and 120 minutes of reperfusion (29.4% ± 2.0% vs. 22.2% ± 1.4%, *n* = 12, **P < 0.01). (**B**) Infarction area plotted against RZ area measured on heart slides as described in Methods confirming the decreased susceptibility to ischemic injury in KI mice. *P* < 0.05 vs. WT. Fluorescence polarization titration revealing the displacement of fluorescein-labeled MKK3b by unlabeled MKK3b (**C**) or TAB1 (**D**). One-way ANOVA and 1-way ANCOVA used.

We also observed that, in the KI hearts, following 30 minutes of regional ischemia and 120 minutes of reperfusion, myocardial infarction volume as a percentage of the risk volume was significantly reduced (Figure 9, A and B). We hypothesize that the protective phenotype we observed in the KI hearts can be associated, at a molecular level, to the abolition of TAB1 phosphorylation.

### A possible compensatory mechanism to explain p38α activation during myocardial ischemia in TAB1 (V390A, Y392A, V408G, and M409A) KI mice

A caveat with our genetic approach is that we stopped TAB1 from docking onto p38α in all circumstances, but by doing so, MAP2K3 and MAP2K6, the archetypical upstream activators of p38α, may have unhindered access to p38α, possibly participating in a compensatory mechanism that could explain the activation of p38α observed in the KI mice. For example, the interaction points of MKK3b, the most abundant splice variant of MAP2K3, on p38α have previously been described and partially overlap with those of TAB1 (8, 20, 21). We therefore examined if TAB1 and MKK3b peptides can compete for access to p38α based on the polarization of fluorescence of C-terminal labelled MKK3b peptide (Figure 9, C and D). From this, it is clear that labeled MKK3b peptide is displaced by TAB1 peptide and that the affinity of TAB1 for p38α is at least as great as that of unlabeled MKK3b peptide. We conclude that TAB1 and MKK3b bind competitively to p38α and that, by preventing TAB1 from docking onto p38α under all circumstances, we may have freed access for MKK3b to p38α.

### Targeting the noncanonical site of p38α-TAB1 interaction with small molecules

**Figure 10:**
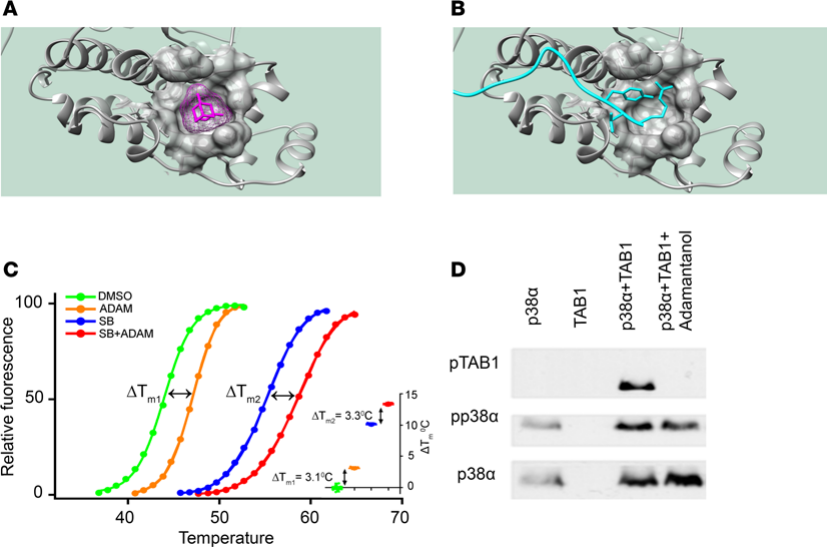
TAB1 and 3-amino-1-adamantanol compete for the same hydrophobic pocket on the noncanonical site of p38α. (**A**) 3-Amino-1-adamantanol binding site. The hydrophobic pocket on p38α is shown as a semitransparent surface, the 3-amino-1-adamantanol molecule is shown in magenta. The hydrogen bonds between the 3-amino group and the hydroxyl group of the adamantane and the oxygens of the backbone carbonyl of Leu 222 and Leu 234 are shown as dotted lines. The van der Waals surface of the adamantanol is shown as a mesh in magenta. (**B**) Zoom-in of the noncanonical site in pp38α-TAB1 complex, the hydrophobic pocket used by the 3-amino-1-adamantanol is shown in identical representation and orientation as in **A**. TAB1 is shown in ribbon form (cyan) with the side chains of Arg 384, Val 385, and Tyr 386 shown. (**C**) Representative fluorescence based thermal shift assay of p38α (5 μM) with DMSO baseline control (green) vs. 12.5 mM 3-amino-1-adamantanol (orange) and DMSO + 50μM SB220025 (blue) vs. 12.5 mM 3-amino-1-adamantanol + 50μM SB220025 (red). Inset: Box and whisker representation indicating that 3-amino-1-adamantanol binding is not competitive to the high-affinity ATP competitive inhibitor SB220025. (**D**) Western blot analysis of pp38α-TAB1 IVKA in the presence and in the absence of 12.5 mM 3-amino-1-adamantanol. It shows that the ligand prevents pp38α phoshorylation of TAB1.

As part of a concerted fragment screen, we identified a group of functionalized adamantanes that bound to one of the hydrophobic pockets on the surface of p38α that forms the NCS for TAB1. Adamantanes consist of 3 fused cyclohexanes that form a stable cage where almost any carbon can be functionalized. We focused our investigations on the 3-amino-1-adamantanol (Figure 10A). The X-ray structure of the complex revealed that the 3-amino and 1-oxydryl group of the adamantane formed hydrogen bonds with the oxygens of the backbone carbonyl of leucine 222 and 234, respectively. The rest of the cage also had a good surface complementarity with the aforementioned hydrophobic pocket (Figure 10B). To validate this crystallography data in solution, we ran a fluorescence-based thermal shift assay with p38α. The thermal shift assay showed that the adamantate stabilizes the kinase (ΔT_m_ = 3.1 °C), relative to DMSO control. In addition, this binding was independent of the ATP-binding pocket, since the stabilization caused by 3-amino-1-adamantanol in the presence (ΔTm_1_ = 3.1 °C) or in the absence (ΔTm_2_ = 3.3 °C) of SB220025, a high-affinity ATP competitive inhibitor, was similar (Figure 10C). We then ran an in vitro kinase assay with pp38α and TAB1 in the presence and in the absence of 3-amino-1-adamantanol, and we show that the ligand is able to interfere with the ability of pp38α to phosphorylate TAB1 (Figure 10D).

## Discussion

We have created a KI mouse harboring 4 residue substitutions in TAB1 that prevent docking onto p38α. The effect is a reduction in myocardial ischemic injury following coronary artery occlusion, despite only minimal reduction of p38α activation. We also observed an almost complete abolition of TAB1 phosphorylation during cardiac ischemia, confirming that the disruption of the p38α-TAB1 complex occurs in vitro. Our hypothesis is that, at a molecular level, the association between TAB1 and p38α drives ischemic damage.

A tool compound that is able to disrupt the interaction between p38α and TAB1 would enable studies to be extended to other species and organs, while permitting temporal control. To this end, our structural data provide a deep understanding of the interaction between the 2 proteins both in their active and nonactive states. The main structural finding is that phosphorylation does not perturb the surface nor the strength of their interaction, suggesting that pTAB1 remains bound to active p38α. Based on our data, we can be confident that any tool compound targeting the surface of the interaction would be active, regardless of the phosphorylation state of the proteins. We identified the adamantanes as binders of the noncanonical site on the kinase and showed that they inhibit TAB1 phosphorylation in vitro. This careful analysis of the p38α surface involved in the interaction supports the concept that the p38α-TAB1complex can be targeted by small molecules, which could ultimately form the basis of a novel class of protein-protein inhibitor (PPI) molecules.

Finally, the genetic model shows that inhibiting the interaction between p38α and TAB1 throughout development is not lethal, suggesting that drugging the interaction will not have the liabilities associated with ATP-competitive inhibitors of p38α kinase activity. The cardioprotection we observed in our KI mouse model and the fact that all of the binding affinity between the 2 proteins was concentrated on a defined surface (the CS and NCS on p38α and a stretch of 29 amino acids on the C-terminal region of TAB1) encourages the development of PPI molecules to recapitulate the benefits of genetic inhibition.

## Methods

### Protein expression and purification.

DNA encoding mouse p38α was derived from a pET14b vector donated by Y. Wang (University of California, Los Angeles) and subcloned into a pETDuet-1 vector. The full coding sequence included an N-terminal His_6_ tag followed by a TEV-cleavage site and mouse p38α sequence. The vector was transformed in *E. coli* strain Rosetta II cells (Novagen). To produce milligram quantities for the structural studies of dual phosphorylated p38α, we ran a large-scale in vitro kinase activation using recombinant constitutively active mkk6DD. The activated p38α was them purified from the reaction mixture, and mass spectrometry was used to confirm stochiometrical phosphorylation of p38α. The protein expression, in vitro activation, and purification procedures followed are described in refs. 22 and 23.

Chemically synthesized DNA encoding mouse TAB1 was subcloned into a pETDuet-1 vector. The full coding sequence included an N-terminal His_6_ tag followed by a TEV-cleavage site and mouse TAB1 (residues 1–438). The vector was transformed in *E. coli* strain Rosetta II cells. Recombinant TAB1 (residues 1–438) was expressed and purified according to the same protocols used for p38α.

To overexpress and purify p^S423^TAB1 (residues 1–438), we followed ref. 24. Briefly, C321(DE3)ΔserB *E. coli* cells were transformed with the pCDF vector containing p^S423^TAB1 (residues 1–438), recovered with 1 ml super optimal broth (SOB) medium, and plated on a streptomycin/chloramphenicol agar plate. The protocols to grow, express, and purify p^S423^TAB1 (residues 1–438) are the same as the ones used for TAB1 and p38α; the only difference is that the cells were grown in the presence of 2 mM O-phospho-L-Serine.

### ITC.

All ITC experiments were carried out on an iTC200 microcalorimeter from Microcal (GE Healthcare). The integrated heat data for the titrations corrected for heats of dilution were fitted with a nonlinear least-squares minimization algorithm to a theoretical titration curve, with MicroCal-Origin 7.0 package. ΔH° (reaction enthalpy change in kcal mol^−1^), Kb (equilibrium binding constant in M^−1^), and n (molar ratio between the 2 species in the syringe and calorimetric cell) were the fitting parameters. The reaction entropy was calculated with the relationships ΔG = −RT × lnKb (R =1.987 cal mol^−1^ K^−1^, T=298 K) and ΔG = ΔH − TΔS, where ΔH represents molar enthalpy change, ΔS represents molar entropy change, and ΔG represents molar Gibbs free energy change..

### X-ray crystallography.

TAB1 and pp38α proteins were purified in 20 mM Tris pH 7.5, 100 mM NaCl, and 10 mM MgCl2 buffer and concentrated to 10 and 20 mg/ml, respectively. The 2 proteins were mixed in the molar ratio of 1.05 TAB1:1.0 pp38α and ATP-γ-S (Tocris Bioscience) added to 10 mM; the final concentration of the complex was 8 mg/ml. Crystallisation screens were set up using a Mosquito dispensing system and a single hit condition obtained (#B12-Proplex screen: 0.1 M MgCl2, 0.1 M Na HEPES pH7.0 and 15% PEG4000, Molecular Dimension). In-house testing showed the crystals were not cryoprotected and diffracted weakly to approximately 3.5 Å. Grid optimizations of PEG/buffer percentage and type were set up together with additive screens; resultant crystals were subjected to various cryoprotection strategies, flash frozen in liquid nitrogen, and tested at Diamond Light Source

Final, optimized crystals were grown from 20% 1:1 PEG4000/PEG6000, 50 mM MgCl2, 50 mM MgSO4, and 0.1 M Tris pH 8.0 and were cryoprotected by rapid transfer through well solution supplemented with 20% ethylene glycol and diffracted to 2.6 Å.

Initial screening of crystals was carried out in-house using an Xcalibur Nova rotating copper anode system with Onyx CCD. Final data were collected at the Diamond synchrotron, beamline IO4.1, 25 Hz Pilatus 6M-F detector. Data were integrated and scaled using Aimless (25), solved by molecular replacement using Phaser (26) with PDBs 4LOO (p38α) and 2J4O (TAB1). The resultant model was refined to completeness by 5 alternating cycles of manual rebuilding in Coot (27) and refinement with Refmac (28). Final statistics and the coordinates have been deposited in the Brookhaven Protein DataBank, PDB code 5NZZ (https://www.rcsb.org/structure/5NZZ).

### Cell biology.

pETDuet-1 vector was used for bacterial coexpression of p38α and the different TAB1 mutants: TAB1 (residues 1–502), TAB1 (residues 1–502) with noncanonical site mutations V390A and Y392A, TAB1 (residues 1–502) with canonical site mutations V408G and M409A, and p38-TAB1 (residues 1–502) with canonical and noncanonical site mutations V390A, Y392A, V408G, and M409A. In all the bacterial vectors, p38α was subcloned in the first multiple-cloning site, whereas TAB1 was subcloned in the second multiple-cloning site.

### Liposome-mediated transfection of HEK293 cells.

HEK293 cells were transfected with plasmids expressing WT p38α, WT TAB1, mutant forms of TAB1, WT TAK1, and WT MKK3. Cells were placed into serum free media (SFM) prior to transfection with complexes containing Turbofect reagent (Thermo Fisher Scientific, catalog R0531) and plasmid DNA. Transfection complexes were prepared and allowed to combine at room temperature for 20 minutes and were then made up to a volume of 1 ml and added drop-wise to cells. Transfection complexes were left on cells overnight and then aspirated and replaced with SFM. Cells were harvested 24 hours after transfection.

### TAB1 (V390A, Y392A, V408G, and M409A) KI mouse generation.

The targeting vector 5′ homology arm and 3′ homology arm was amplified from BAC DNA and confirmed by end sequencing; the mutations V390A, Y392A, V408G, and M409A were introduced into exon 10 by site-directed mutagenesis with QuikChange Site-Directed Mutagenesis Kit (Agilent Technologies). The targeting construct was electroporated into C57BL/6 ES cells, 375 G418-resistant colonies were picked, and the PCR assays were performed with primers 5PCR_F and 5PCR_R. Thirty-four potential targeted clones were identified. Six of them were expanded and frozen. The Southern blot analysis was conducted with 5′-probe, 3′-probe, and Neo-probe. The genomic DNA of the potential clones were digested with Bg1II for the 5′-probe and analyzed by Southern blot for a 8.0-Kb band from the WT allele and a 10.0 Kb band for the recombinant allele; for the 3′ probe, the genomic DNA was digested with XmaI and analyzed by Southern blot for a 7.8-Kb band from the WT allele and a 5.7-Kb band for the recombinant allele. Regarding the Neo probe, the genomic DNA was digested with NsiI and analyzed by Southern blot for a 6.6-Kb band for the recombinant allele.

TAB1 (V390A, Y392A, V408G, and M409A) F1 mice were generated from the ES cell lines 2C9. Five (3 males and 2 females) out of 8 pups were identified as positive by PRC screening: the expected 261-bp fragment from WT allele and 405-bp fragment from recombinant allele were represented as positive F1 mice. The primers used for the screening were: Neo_Del_F and Neo_Del_R.

The primers sequences used were: 5PCR_F: 5′-GCGGAGTATTAGGAGCCTGAGGGT-3′, 5PCR_R: 5′-GCTGACCGCTTCCTCGTGCTTTA-3′, 5′-probe_F: 5′-CAGCCACTTGTGAGACCAGAGGA-3′, 5′-probe_R: 5′-GGACAAGCCTGTGAGGTGATGACA-3′, 3′-probe_F: 5′-GGTCCCTCACAAGGGTTAAGCAACT-3′, 3′-probe_R: 5′-GAACTTGTCTGTGCTCTGAGCTGGC-3′, Neo-probe_F: 5′-CCTGAATGAACTGCAGGACGAGG-3′, Neo-probe_R: 5′-AGCTCTTCAGCAATATCACGGGTAGC-3′, Neo_Del_F: 5′-GCTGGCCTTGCTCAACTCCAG-3′, Neo_Del_R: 5′-GACCATCTGTCTCATACCTGACCTCAC-3′.

### Library preparation and next-generation sequencing.

RNA isolated from the 10 left ventricles (5 for each condition: WT vs. KI) was quantified using Qubit fluorometric quantification and assessed for quality using Agilent Bioanalyzer. Library preparation was performed using NEBNext Ultra Directional RNA Library Prep Kit for Illumina and the NEBNext rRNA Depletion kit (New England BioLabs). The library was sequenced on an Illumina HiSeq2500 at the Genomic Centre at King’s College London (paired-end, 2 × 125 bps, >25 M reads per samples, HiSeq SBS Kit V4).

### RNA-Seq data analysis.

RNA-Seq data of mouse heart cell populations were obtained as paired-end reads. Each sample contained typically 10–11 million reads. Sequence reads were aligned to the Ensembl GRCm38 genome using TopHat2 (29); alignment rates were 82.9% ± 1.9%. Transcripts were counted using the featureCounts program. Data analysis was performed in the R environment (R Core Team 2016). The matrix of merged transcript counts (30) was processed with a quality filter to remove genes with no counts in any cell sample. The dimension of the final data matrix was 14,864 genes by 10 samples. The entire RNA-Seq data flow was managed by a GNU make pipeline. Transcript counts were further analyzed for differential gene expression using the DESeq2 package (31).

The RNA-Seq data have been deposited in Annotare 2.0 webserver, https://www.ebi.ac.uk/fg/, entry code: E-MTAB-6460

### Immunophenotype.

Single cell suspensions from spleen were prepared by mechanical disruption using Miltenyi C-tubes, followed by enzymatic digestion in PBS Ca^2+^/Mg^2+^, 2% FCS (v/v), 10 mM HEPES, Collagenase (1 mg/ml), and DNAse (0.1 mg/ml) 37°C for 30 minutes. Cells were isolated from mesenteric lymph nodes using manual mechanical disruption followed by enzymatic digestion (as above) for 15 minutes at 37°C. BM immune cells were isolated from the femur by centrifugation (5,000 *g*). Spleen and BM RBCs were lysed using RBC lysis buffer (eBioscience) for 90 seconds at room temperature. All samples were incubated with FC block (clone 2.4G2, BD Biosciences) for 10 minutes at 4°C prior to staining with live/dead dye (Zombie NIR; BioLegend) for 10 minutes at room temperature. Samples were then stained with the following antibody panels at 4°C for 20 minutes. T cell panel; CD45 Qdot 605, CD5 BV510, TCR-d Pe-Cy7, CD161/NK1.1 BV650, CD4 BV786, CD8 AF700, CD25 APC, GITR PE, CD44 FITC, CD62L PerCP-Cy5.5, KLRG1 BV421. Myeloid panel; CD45 Qdot 605, CD11c BV786, CD11b BV510, F4/80 PerCP-Cy5.5, Ly6C AF700, Ly6G APC, CD103 PE, CD317 BV650, MHCII/IA/IE FITC, CD86 PE-Cy7, (lineage) CD3, CD19, NK1.1 BV421. B Cell Panel; CD45 Qdot, IgG1 PE, B220 (CD45R) AF-700, IgM BV786, IgD PerCP-Cy5.5, GL-7 AF647, CD95 PE-Cy7, CD138 BV650, CD5 BV510, CD21/35 FITC, CD23 BV421. NK/myeloid panel; CD19 Pe-Cy7, CD5 APC, Ly6G APC, Ly6C AF700, MHCII/IA/IE FITC, CD11c BV786, CD161/NK1.1 BV650, CD21/35 PE, CD11b BV510, CD23 BV421. Bone marrow panel; CD45 Qdot 605, CD43 PerCP-Cy5.5, GR1 AF700, CD11b BV510, B220/CD45R Pe-Cy7, CD24 APC, CD138 BV650, BP1/Ly51 PE, CD3 BV785, IgD AF488, IgM BV421 (antibody information is detailed in Supplemental Table 1; supplemental material available online with this article; https://doi.org/10.1172/jci.insight.121144DS1). Samples were acquired using a BD Fortessa X20 equipped with 405 nm, 488 nm, 561 nm and 644 nm lasers. Data was analyzed using DIVA software (BD Biosciences), and plots were produced in R using the ggplot2 package.

### In vivo ischemia/reperfusion and measurement of the myocardial infarction volume as a percentage of the risk volume.

Animal studies used age-matched (12–14 weeks old) WT and TAB1 KI male mice (Cyagen Biosciences). Mice were placed inside an induction chamber, where 4% isoflurane was provided with an oxygen flow rate of 0.5 l/min until loss of righting reflex. Anesthetized mice on a vertical support suspended by its upper incisors were intubated by inserting a 20-G cannula through the trachea. Artificial ventilation of the lungs was provided with a mouse respirator (Hugo Sachs Elektronic MiniVent Type 845) set at 200 μl of stroke volume and at 150 strokes per minute venting 2% isoflurane. A rectal probe was inserted to monitor and to adjust the homeothermic warming pad to maintain the temperature at 37°C ± 1°C. The thorax was exposed and an incision was performed in the third intercostal space. Then, the operating region was exposed by using chest microretractors. Temporary ischemia of the left ventricle was achieved by placing a 0.2 cm PE90 tube parallel to the left anterior descending coronary artery (LAD) and looping an 8-0 nylon suture (Ethilon, Johnson & Johnson) around both tube and LAD. The PE90 tube and the knot were released 30 minutes after ischemia, leaving a loose suture in the area where the occlusion took place. Reperfusion was confirmed visually by the return of blood flow back into the myocardium. To assess the area at risk, the loose suture left around the LAD was tightened after 2 hours of reperfusion, and 300 μl of 3% Evans Blue (3% w/v in 0.9% v/w NaCl) was injected in the left ventricle via the apex to delineate the risk zone (RZ). Heart samples were collected by excising and snap-freezing the whole heart, which was then cut into 750-μm sections from apex to base using a custom mouse block. All slices were incubated at 37°C for 20 minutes with 3% w/v 2,3,5-triphenyltetrazolium chloride (MilliporeSigma) dissolved in 0.1 M Na_2_HPO_4_/NaH_2_PO_4_ buffer adjusted to pH 7.4. Slices were fixed overnight in 10% formaldehyde and then placed between 2 cover slips and digitally imaged using a high-resolution optical scanner (Epson). Images were analyzed using ImageJ (SciJava), and the size of infarcted area, left ventricle area at risk, and normally perfused left ventricle zone were outlined in each section by identification of their appearance and borders. Areas were quantified on both sides of each slice and averaged. Infarct size (INZ) was calculated as a percentage of RZ for each heart. To produce ischemic hearts for the biochemical analysis, permanent ischemia was performed by directly tightening the LAD with a double knot; heart samples were collected by cutting and snap-freezing the apex 5 and 10 minutes after occlusion.

### Western blot analysis of p38α, TAB1, pp38α, and pTAB1 in ischemic heart homogenate.

Rapidly snap-frozen apex samples were homogenized in a cold glass pestle and mortar tube containing chilled buffer (50 mM Tris-HCl pH 7.5, 1 mM EDTA, 1 mM EGTA, 1% Triton X100, 0.1 % β-mercaptoethanol, 1 mM Na_3_VO_4_, 50 mM NaF, 5 mM Na_4_P_2_O_7_, and 1 protease inhibitor cocktail tablet per 50 ml [cOmplete, Roche Diagnostics]). Homogenates were centrifuged (16,000 *g*) at 4°C for 10 minutes, and supernatants were collected and added to 2× SDS buffer (20% glycerol, 6% SDS in 0.12 M Tris pH 6.8, 10% 2-mercaptoethanol, and 0.4% Bromophenol blue) and boiled for 10 minutes. Samples were resolved on 10% SDS PAGE gels under denaturing conditions and transferred onto PVDF membranes. After blocking in 4% nonfat milk and 1% BSA in Tris-buffered saline, pH 7.4, for 1 hour, membranes were exposed to the following primary antibodies overnight at 4°C, with agitation: anti–dual phospho-p38 (Thr^180^ and Tyr^182^, 9211, Cell Signaling Technology), total p38 (9212, Cell Signaling Technology), anti–phospho TAB1Ser423, and total TAB1 (both from MRC PPU reagents, S739A, S823A). After washing and incubation with HRP-conjugated secondary antibody, antigen-antibody complexes were visualized by enhanced chemiluminescence detection (Pierce Biotechnology).

### MKK3 competitive binding assay.

A titration of TAB-1 (Ac-RVYPVSVPYSSAQSTSKTSVTLSLVMPSQ-NH_2_) or MKK3b (Ac-GKSKRKKDLRISCMSKP-NH_2_) peptide was dispensed from a concentrated solution in DMSO using a Hewlett Packard D300 digital dispenser directly into a 384-well low-volume assay plate. The DMSO concentration was normalized to 1% of the final assay volume. To this was added a mixture of 2 nM fluorescein-labeled MKK3b peptide (Ac-GKSKRKKDLRISCMSKPK[ε-fluorescein]-NH_2_) and 10 μM p38α in a buffer of 50 mM HEPES pH 7.4, 10 mM MgCl2, 1 mM Chaps, and 1 mM DTT. The final well volume was 10 μl. After incubation at ambient temperature for 20 minutes, fluorescence polarization was measured on a Perkin Elmer multimode reader (480 nm excitation filter, 535 nm emission filters, 505 nm mirror). Data were normalized between averaged control wells of DMSO only (0% displacement) and ligand only (100% displacement). Duplicate titrations were averaged and fitted to a 4-parameter IC_50_ function.

### Thermal shift assays.

Thermal shift assays were performed using an Agilent MX3005p quantitative PCR (qPCR) instrument with 70 cycles at 1°C interval from 24°C–94°C. Samples were prepared by mixing recombinant purified p38α with buffer (40 mM Tris pH7.5, 100 mM NaCl, and 4 mM DTT) and ligands ± DMSO to normalize the DMSO level to 2.5% in all samples. Samples were incubated at room temperature for 30 minutes prior to assays. Sypro Orange (Invitrogen, S6651, 5,000× concentrate in DMSO) was separately prepared at 1:1,000 dilution in the same buffer and then mixed 1:1 with the protein/ligand samples just prior to the thermal shift assays. The final concentration in the assays was 5 μM p38 ± 12.5 mM 3-amino-1-adamantanol (Fluorochem ) ± 50 μM SB220025 (MilliporeSigma). Melting curves were analyzed using PRISM and Tm values calculated using the Boltzmann sigmoidal function. Each experiment was performed 9 times, and mean ΔTm values calculated for DMSO vs. 3-amino-1-adamantanol (ΔTm_1_) and SB220025 vs. SB220025 + 3-amino-1-adamantanol (ΔTm_2_).

### In vitro kinase assay.

An in vitro kinase assay was performed with recombinant active p38α and TAB1 (residues 1–438), and 3 μM of pp38α was incubated with 15 μM of TAB1, with or without 12.5 mM 3-amino-1-adamantanol. The reaction was carried out in 1× kinase buffer (25 mM Tris/HCl pH 7.5, 5 mM β-glycerolphosphate, 2 mM DTT, 0.1 mM Na_3_VO_4_, and 1 mM MgCl_2_). ATP (550 μM) was added to the incubation mixture to start the reaction at 37^o^C for 2 hours. At the end of reaction, the samples were collected with 2× sample buffer (20% glycerol, 6% SDS in 0.12 M Tris pH6.8, 10% 2-mercaptoethanol, and 0.4% Bromophenol blue), heated to 95°C for 10 minutes, and run on 10% SDS gel under denaturing conditions. The samples were transferred onto a PVDF membrane using semidry technique, blocked with 4% milk + 1% BSA for 1 hour at room temperature, and probed with appropriate primary antibody overnight at 4°C. The blots were probed with appropriate secondary antibody linked with HRP and visualized by enhanced chemiluminescence. Antibodies used included anti–dual-phosphorylated p38 (Thr180/Tyr182) (M8177, MilliporeSigma; 9211, Cell Signaling Technology) at 1:5,000, total p38 (9212, Cell Signaling Technology) at 1:10,000, anti–phosphorylated TAB1 (Phil Cohen group, University of Dundee) at 1:2,000.

### Statistics.

The statistical analysis used when comparing multiple groups was 1-way or 2-way ANOVA, as indicated in the figures legend. For repeated measures when comparing multiple measurements within subjects (Figure 7, C and D), the Bonferroni correction was used to adjust the threshold for statistical significance. A 1-way ANCOVA test was used for Figure 9B. *P* < 0.05 was considered significant.

### Study approval.

This investigation was performed in accordance with the Home Office Guidance on the Operation of the Animals (Scientific Procedures) Act 1986, published by Her Majesty’s Stationery Office (London, United Kingdom). Animals were maintained humanely in compliance with the “Principles of Laboratory Animal Care” formulated by the National Society for Medical Research and the Guide for the Care and Use of Laboratory Animals (*National Academies Press*, 2011). All animal protocols were approved both by the local King’s College Animal Welfare and Ethical Review Board and by the UK Government Home Office (Animals Scientific Procedures Group).

## Author contributions

GFDN cowrote the manuscript, supervised the work, designed and performed the biophysical experiments, and expressed and purified the recombinant proteins. RB helped run the mice infarction studies and ran the Western blots of the mice hearts and of the in vitro kinase assay. CN ran the X-rays and thermofluor experiments and expressed and purified the recombinant proteins. MFC ran the mouse infarction studies. PAG ran the cell biology experiments. DT, RA, EDM, and SV helped maintaining the mouse colony. JK ran the computational analysis of the RNA-Seq data. AL ran the immunophenotype experiments. JPH run the fluorescence polarization experiments. PE helped with the Discussion section. JC ran the analysis of the mice infarction data. MSM cowrote the manuscript and supervised the work.

## Supplementary Material

Supplemental data

## Figures and Tables

**Table 2:**
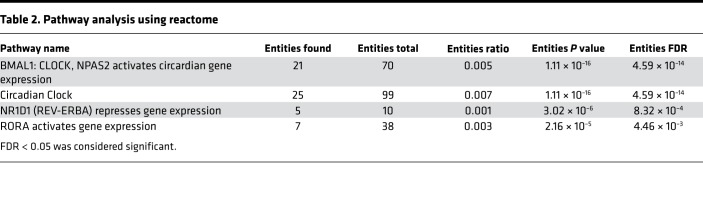
Pathway analysis using reactome

**Table 1:**
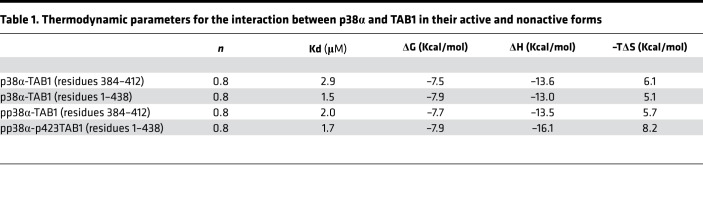
Thermodynamic parameters for the interaction between p38α and TAB1 in their active and nonactive forms

## Version 1 08/23/2018

Electronic publication
