# Supplementary material for: The TAB1-p38α complex aggravates myocardial injury and can be targeted by small molecules
Source: JCI Insight. 2018 Aug 23;3(16):e121144. doi: 10.1172/jci.insight.121144 (PMC6141180; doi:10.1172/jci.insight.121144)
Supplement: Supplemental data [file jciinsight-3-121144-s200.pdf]

REAGENT or RESOURCE	SOURCE	IDENTIFIER
Antibodies (epitope)		
CD3 ε BV421	BD	562600
CD3 ε BV785	Biolegend	100232
CD4 BV786	BD	563331
CD5 APC	BD	550035
CD5 BV510	BD	563069
CD5 BV510	BD	563069
CD8 α AF700	BD	557959
CD11b BV510	Biolegend	101245
CD11b BV510	Biolegend	101245
CD11c BV786	BD	563735
CD19 BV421	BD	562701
CD19 PECY7	BD	552854
CD21/35 FITC	BD	553818
CD21/35 PE	BD	552957
CD23 BV421	BD	562929
CD23 BV421	BD	562929
CD24 APC	BD	562349
CD25 APC	BD	557192
CD43 PerCP-Cy5.5	Biolegend	121224
CD44 FITC	BD	553133
CD45 e450	eBioscience	48-0451-82
CD45 eVolve™ 605 (Qdot)	eBioscience	83-0451-42
CD45 eFluor450	eBioscience	Cat# 48-0451-82
CD45R (B220) PeCY7	Biolegend	103222
CD45R (B220) AF700	BD	557957
CD62L PerCP Cy5.5	BD	560513
CD86 PECY7	BD	560582
CD95 PECY7	BD	557653
CD103 PE	BD	557495
CD138 BV650	Biolegend	142517
CD138 BV650	Biolegend	142517
CD161 (NK1.1) BV421	BD	108732
CD161 (NK1.1) BV650	Biolegend	108736
CD317 BV650	Biolegend	127019
F4/80 PerCP-Cy5.5	Biolegend	123128
Fc block	BD	553142
GITR PE	BD	558119
GL-7 AF647	BD	561529
Gr-1 AF700	Biolegend	108422
Ig	Dako	P0447
IgD PerCP-Cy5.5	Biolegend	405710
IgD AF488	Biolegend	405718
IgG1 PE	BD	550083
IgM BV42	Biolegend	406518
IgM BV786	BD	564028

KLRG1 BV421	BD	562897
Ly6C AF700	BD	561237
Ly6G APC	BD	560599
MHCII IA/E FITC	BD	553623
